# Supplementary material for: Head-to-Head Comparison of Nasopharyngeal, Oropharyngeal and Nasal Swabs for SARS-CoV-2 Molecular Testing
Source: Diagnostics (Basel). 2023 Jan 12;13(2):283. doi: 10.3390/diagnostics13020283 (PMC9857511; doi:10.3390/diagnostics13020283)
Supplement: Supplementary file 1 [file diagnostics-13-00283-s001.zip › diagnostics-2070730-supplementary.pdf]

Supplementary Table S1. Inclusion site, RT-PCR system and kits.

Record ID	Inclusion site	RT-PCR system
1	Rigshospitalet	Pentabase CoviDetect™
2	Hillerød	Agilent ArixMx Real-time PCR System
3	Rigshospitalet	Pentabase CoviDetect™
4	Hillerød	Aptima™ SARS-CoV-2 Assay (Panther™ System)
5	Køge	Allplex™ SARS-CoV-2 real-time PCR assay
6	Køge	Allplex™ SARS-CoV-2 real-time PCR assay
7	Køge	Allplex™ SARS-CoV-2 real-time PCR assay
8	Køge	Allplex™ SARS-CoV-2 real-time PCR assay
9	Køge	Allplex™ SARS-CoV-2 real-time PCR assay
10	Køge	Allplex™ SARS-CoV-2 real-time PCR assay
11	Rigshospitalet	Pentabase CoviDetect™
12	Hillerød	2019-nCoV real-time fluorescent RT-PCR kit - BGI
13	Køge	Allplex™ SARS-CoV-2 real-time PCR assay
14	Rigshospitalet	Pentabase CoviDetect™
15	Rigshospitalet	Pentabase CoviDetect™
16	Hillerød	Aptima™ SARS-CoV-2 Assay (Panther™ System)
17	Køge	Allplex™ SARS-CoV-2 real-time PCR assay
18	Køge	Allplex™ SARS-CoV-2 real-time PCR assay
19	Rigshospitalet	Pentabase CoviDetect™
20	Hillerød	Aptima™ SARS-CoV-2 Assay (Panther™ System)
21	Køge	Allplex™ SARS-CoV-2 real-time PCR assay
22	Køge	Allplex™ SARS-CoV-2 real-time PCR assay
23	Køge	Allplex™ SARS-CoV-2 real-time PCR assay
24	Køge	Allplex™ SARS-CoV-2 real-time PCR assay
25	Rigshospitalet	Pentabase CoviDetect™
26	Køge	Allplex™ SARS-CoV-2 real-time PCR assay
27	Køge	Allplex™ SARS-CoV-2 real-time PCR assay
28	Rigshospitalet	Pentabase CoviDetect™
29	Rigshospitalet	Cobas 6800 - Cobas® SARS-CoV-2
30	Køge	Allplex™ SARS-CoV-2 real-time PCR assay
31	Køge	Allplex™ SARS-CoV-2 real-time PCR assay
32	Rigshospitalet	Cobas 6800 - Cobas® SARS-CoV-2
33	Køge	Allplex™ SARS-CoV-2 real-time PCR assay
34	Køge	Allplex™ SARS-CoV-2 real-time PCR assay
35	Rigshospitalet	Pentabase CoviDetect™
36	Køge	Allplex™ SARS-CoV-2 real-time PCR assay
37	Rigshospitalet	Cobas 6800 - Cobas® SARS-CoV-2
38	Køge	Allplex™ SARS-CoV-2 real-time PCR assay
39	Rigshospitalet	Cobas 6800 - Cobas® SARS-CoV-2

40	Hillerød	Aptima™ SARS-CoV-2 Assay (Panther™ System)
41	Køge	Allplex™ SARS-CoV-2 real-time PCR assay
42	Køge	Allplex™ SARS-CoV-2 real-time PCR assay
43	Køge	Allplex™ SARS-CoV-2 real-time PCR assay
44	Rigshospitalet	Pentabase CoviDetect™
45	Rigshospitalet	Pentabase CoviDetect™
46	Rigshospitalet	Pentabase CoviDetect™
47	Hillerød	Pentabase CoviDetect™
48	Rigshospitalet	Pentabase CoviDetect™
49	Rigshospitalet	Pentabase CoviDetect™
50	Rigshospitalet	Pentabase CoviDetect™
51	Rigshospitalet	Cobas 6800 - Cobas® SARS-CoV-2
